# MiR-1207 overexpression promotes cancer stem cell–like traits in ovarian cancer by activating the Wnt/β-catenin signaling pathway

**DOI:** 10.18632/oncotarget.4921

**Published:** 2015-08-17

**Authors:** Geyan Wu, Aibin Liu, Jinrong Zhu, Fangyong Lei, Shu Wu, Xin Zhang, Liping Ye, Lixue Cao, Shanyang He

**Affiliations:** ^1^ Department of Obstetrics and Gynecology, The First Affiliated Hospital, Sun Yat-sen University, Guangzhou 510700, PR China; ^2^ State Key Laboratory of Oncology in Southern China, Department of Experimental Research, Sun Yat-sen University Cancer Center, Guangzhou 510060, PR China; ^3^ Department of Biochemistry, Zhongshan School of Medicine, Sun Yat-sen University, Guangdong 510080, PR China

**Keywords:** ovarian cancer, Wnt/β-catenin signaling, cancer stem cells, tumorigenicity

## Abstract

Wnt/β-catenin signaling pathway is strictly controlled by multiple negative regulators. However, how tumor cells override the negative regulatory effects to maintain constitutive activation of Wnt/β-catenin signaling, which is commonly observed in various cancers, remains puzzling. In current study, we reported that overexpression of miR-1207 in ovarian cancer activated Wnt/β-catenin signaling by directly targeting and suppressing secreted Frizzled-related protein 1 (SFRP1), AXIN2 and inhibitor of β-catenin and TCF-4 (ICAT), which are vital negative regulators of the Wnt/β-catenin pathway. We found that the expression of miR-1207 was ubiquitously upregulated in both ovarian cancer tissues and cells, which inversely correlated with patient overall survival. Furthermore, overexpression of miR-1207 enhanced, while silencing miR-1207 reduced, stem cell-like traits of ovarian cancer cells *in vitro* and *in vivo*, including tumor sphere formation capability and proportion of SP+ and CD133+ cells. Importantly, upregulating miR-1207 promoted, while silencing miR-1207 inhibited, the tumorigenicity of ovarian cancer cells. Hence, our results suggest that miR-1207 plays a vital role in promoting the cancer stem cell-like phenotype in ovarian cancer and might represent a potential target for anti-ovarian cancer therapy.

## INTRODUCTION

Ovarian cancer, one of the most lethal gynecologic malignancies, constitutes 4% of all cancers diagnosed in women, with 6.6 new cases per 100,000 women occurring worldwide each year [1, 2]. The 5-year overall survival rate of patients with ovarian cancer is less than 40%, resulting in an estimated 14,030 deaths in 2013 [3]. Although early-stage ovarian cancer can be successfully removed with surgery alone, most patients are diagnosed at the advanced stages. Surgical resection followed by platinum-based chemotherapy has been acknowledged as the standard treatment for ovarian cancer, but most patients ultimately develop recurrence, which leads to insurmountable obstacles for improving the survival rate [4–6]. Cancer stem cells (CSCs), a small subpopulation of cancer cells, which possess self-renewal and multi-lineage differentiation potential, have been reported to be responsible for ovarian cancer progression, such as recurrence [7–9]. Therefore, to unveil the underlying molecular mechanism of ovarian CSCs turn out to be the essential and profound topic for cancer treatment.

The Wnt/β-catenin signaling pathway, one of the most relevant pathways involved with CSCs, has been reported to be aberrantly activated in various types of cancers, including ovarian cancer [10–13]. Previous study showed that multiple downstream genes of Wnt signaling are associated with chemoresistance and recurrence of ovarian cancer [14]. Additionally, Chau et al. reported that c-Kit regulates ovarian cancer stemness through activation of Wnt/β-catenin signaling [15]. However, the underlying mechanism of how Wnt/β-catenin signaling regulating ovarian CSCs is still largely unclear.

In the canonical Wnt/β-catenin signaling pathway, when ligand Wnt engages to the Frizzled receptor (Fz) and the low-density lipoprotein receptor-related protein-5/6 (LRP-5/6), Dishevelled (Dvl) is phosphorylated and consequently releases β-catenin from the destruction complex, formed by Axin, adenomatous polyposis coli (APC), casein kinase 1α (CK1α) and glycogen synthase kinase 3β (GSK-3β) [16, 17]. Thereby, β-catenin translocates into the nucleus and activates downstream target genes involved in CSC regulation alongside the T-cell factor/lymphoid enhancer factor (TCF/LEF) transcription factors. [18]

On the other hand, there are multiple levels of negative modulators involved in the Wnt/β-catenin signaling pathway for fine tuning the signaling. [19, 20]. For instance, as extracellular secreted Wnt inhibitors, secreted Frizzled-related proteins (SFRPs) can suppress ligands binding to Fz and blocking signal transduction [21]. Moreover, Axin associates with GSK-3β, CK1α and APC to form the destruction complex that induces β-catenin degradation through ubiquitin-proteasome pathway [22]. Inhibitor of β-catenin and TCF-4 (ICAT) can bind and inhibit the interaction between β-catenin with the transcription factor TCF [23]. Thus, understanding how these negative regulatory effects on the Wnt/β-catenin signaling pathway, which are concomitantly deregulated in ovarian cancer, is biologically as well as clinically important for future development of ovarian cancer treatment.

MicroRNAs (miRNAs), endogenous non-coding small RNAs, can simultaneously interact with multiple targets at the post-transcriptional level [24]. In the current study, we found that miR-1207 was significantly overexpressed in ovarian cancer and enhanced the stem cell-like traits of ovarian cancer cells by downregulating of multiple negative modulators of the Wnt/β-catenin pathway, including SFRP1, AXIN2 and ICAT. Therefore, our results suggest that miR-1207 might serve as a novel therapeutic target in ovarian cancer.

## RESULTS

### MiR-1207 overexpression correlates with ovarian cancer progression

**Figure 1:**
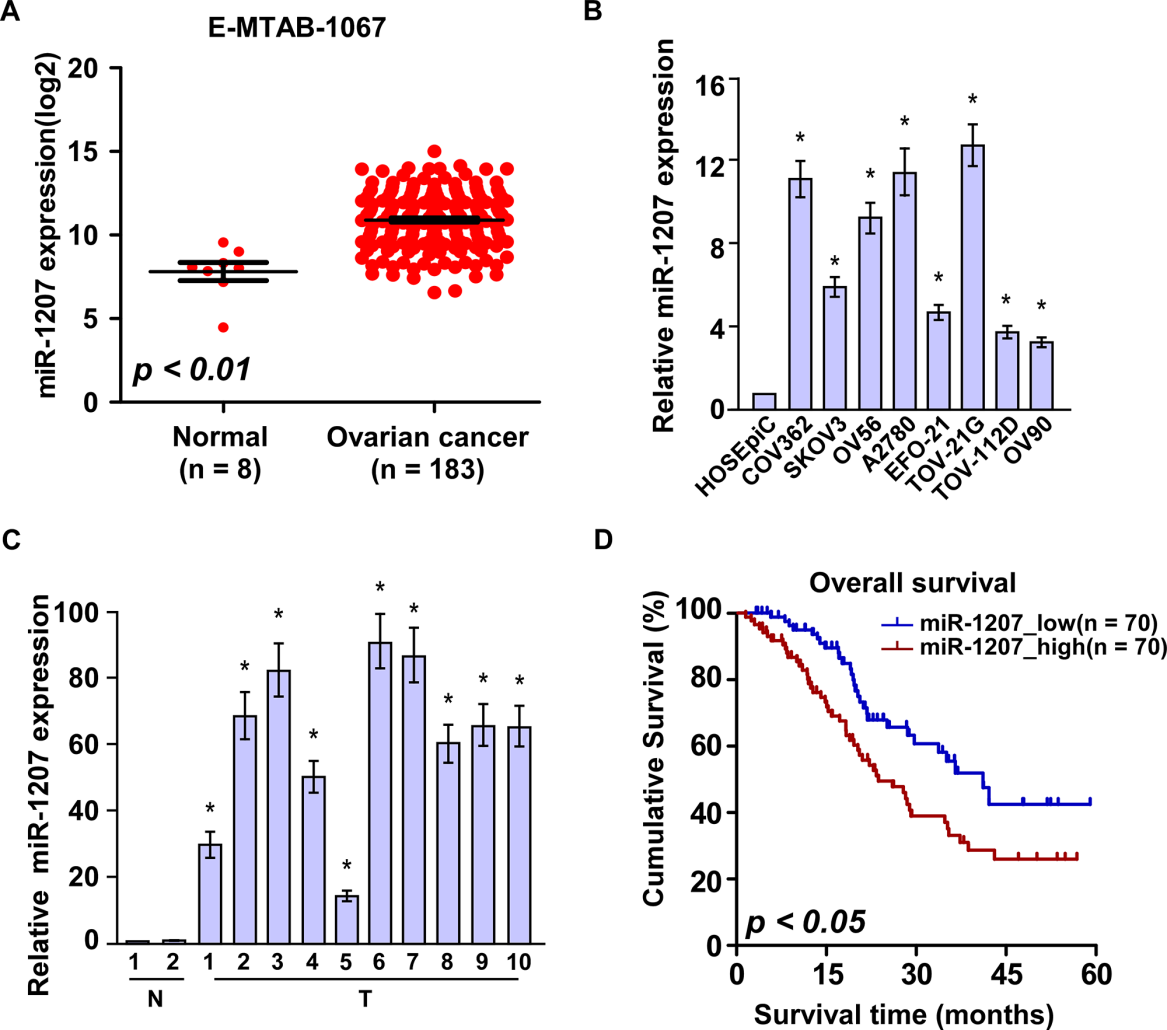
Overexpression of miR-1207 is positively associated with ovarian cancer prognosis **A.** Expression profiling of miR-1207 from published microarray-based high-throughput datasets (*n* = 191; NCBI GEO, E-MTAB-1067). **B–C.** Real-time PCR analysis of miR-1207 expression in eight ovarian cancer cell lines (B) and ten ovarian cancer samples (T) (C) compared to HOSEpiC or normal ovarian tissues (N) Transcript levels were normalized to *U6* expression. Bars represent the mean ± SD of three independent experiments. *P < 0.05. **D.** Overall survival of patients with low versus high expression of miR-1207 (*n* = 140; *P* < 0.05, log-rank test).

By analyzing published miRNA expression profiles obtained from 183 patients of ovarian cancer (E-MTAB-1067), we found that miR-1207 was significantly upregulated in 183 ovarian cancer tissues compared to eight normal human ovarian surface epithelial (HOSE) cell lines (Figure 1A). Furthermore, real-time PCR analysis was performed and showed that miR-1207 was ubiquitously overexpressed in all eight ovarian cancer cell lines and ten ovarian cancer samples compared to human ovarian surface epithelial cells (HOSEpiC) and two normal ovarian-adjacent tissues, respectively (Figure 1B–1C). Collectively, these findings suggest that miR-1207 expression is upregulated in ovarian cancer.

To further explore the clinical relevance of miR-1207 in ovarian cancer progression, we therefore extended the quantification of miR-1207 to a cohort of 140 archived human ovarian cancer specimens (Supplementary Table S1). Statistical analysis revealed that miR-1207 expression was positively correlated with International Federation of Gynecology and Obstetrics (FIGO) stage (*P* < 0.001), lymph node metastasis (*P* < 0.001) in patients with ovarian cancer (Supplementary Table S2). Importantly, patients with higher miR-1207 expression had shorter survival time, whereas patients with lower miR-1207 expression had longer survival time (*P* < 0.05; Figure 1D). Collectively, these results indicate a possible link between miR-1207 overexpression and the progression of human ovarian cancer.

### Upregulation of miR-1207 promotes CSC-like traits in ovarian cancer

**Figure 2:**
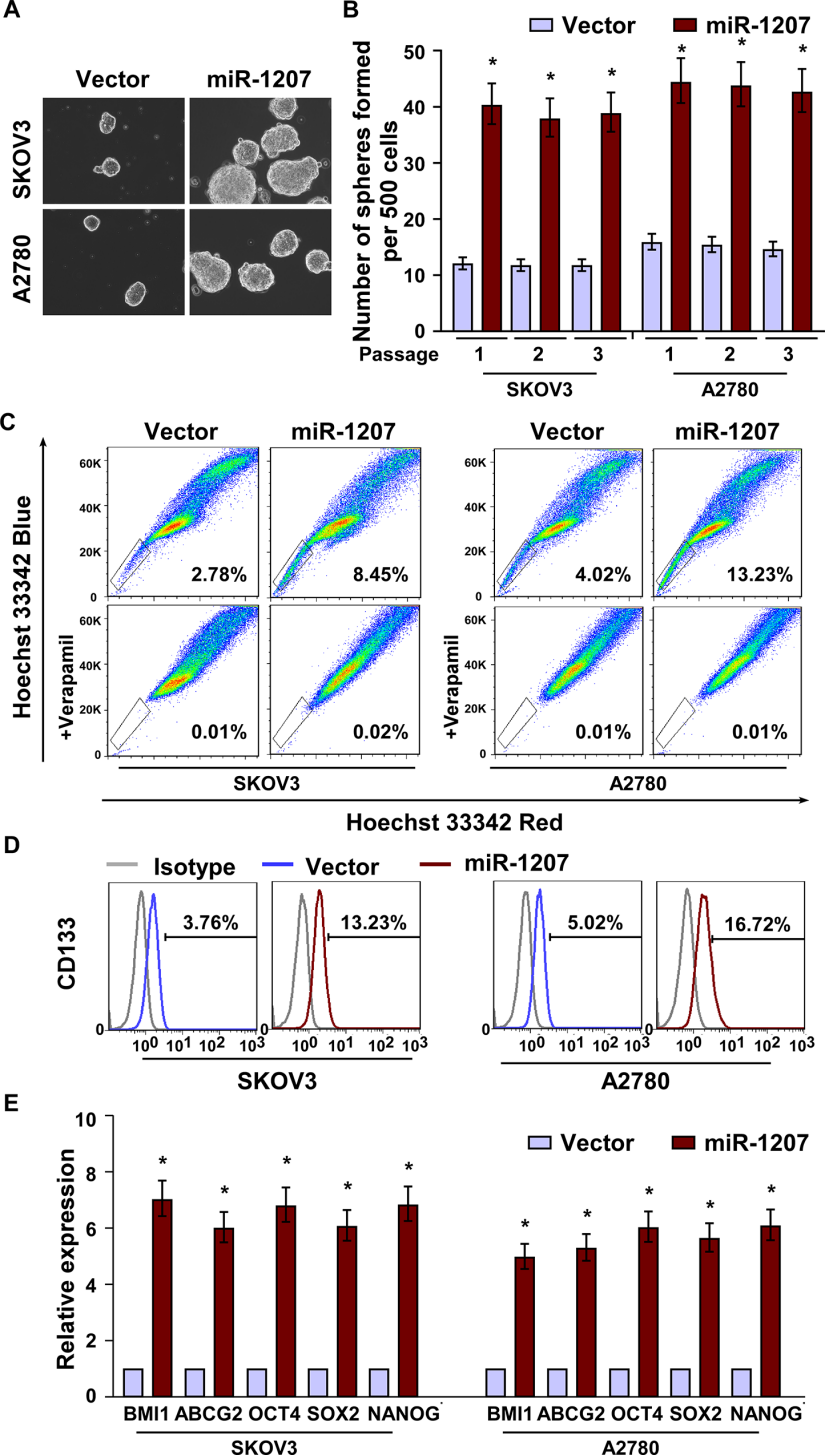
MiR-1207 overexpression promotes cancer stem–like traits in ovarian cancer **A–B.** Representative micrographs (A) and quantification (B) of tumor sphere formation in miR-1207 overexpression or vector cells. Scale bar, 100 μm. **C–D.** Hoechst 33342 dye exclusion assay showing the effect of miR-1207 overexpression cells or vector cells on the distribution of SP^+^ (C) and CD133^+^ cells (D) determined using flow cytometric analysis. **E.** Real-time PCR analysis of mRNA expression of pluripotency-associated markers in the cells. Transcript levels were normalized to *GAPDH* expression. Error bars represent the mean ± SD of three independent experiments. *P < 0.05.

To investigate the pathological role of miR-1207 expression in ovarian cancer progression, we established stable SKOV3 and A2780 cell lines expressing miR-1207 (Supplementary Figure S1A). The tumor sphere formation assay showed that miR-1207–transduced cells formed more and larger spheres than the vector cells (Figure 2A–2B). As side populations (SP) cells have been identified to closely correlate with cancer stem cell-like traits and CD133 expression can define a tumor initiating cell population in primary human ovarian cancer [25, 26], we found that the subpopulations of SP^+^ and CD133^+^ cells were both significantly increased in miR-1207-transduced ovarian cancer cells compared with the vector control cells (Figure 2C and 2D). Moreover, miR-1207 overexpression dramatically upregulated the mRNA expression levels of multiple pluripotency factors, including BMI1 proto-oncogene (BMI1), adenosine triphosphate–binding cassette sub-family G member 2 (ABCG2), POU class 5 homeobox 1 (OCT4), sex-determining region Y-box 2 (SOX2), and Nanog homeobox (NANOG) (Figure 2E). Taken together, our results suggest that upregulation of miR-1207 promotes the stem cell-like traits of ovarian cancer cells.

### Downregulation of miR-1207 suppresses ovarian CSC-like traits

**Figure 3:**
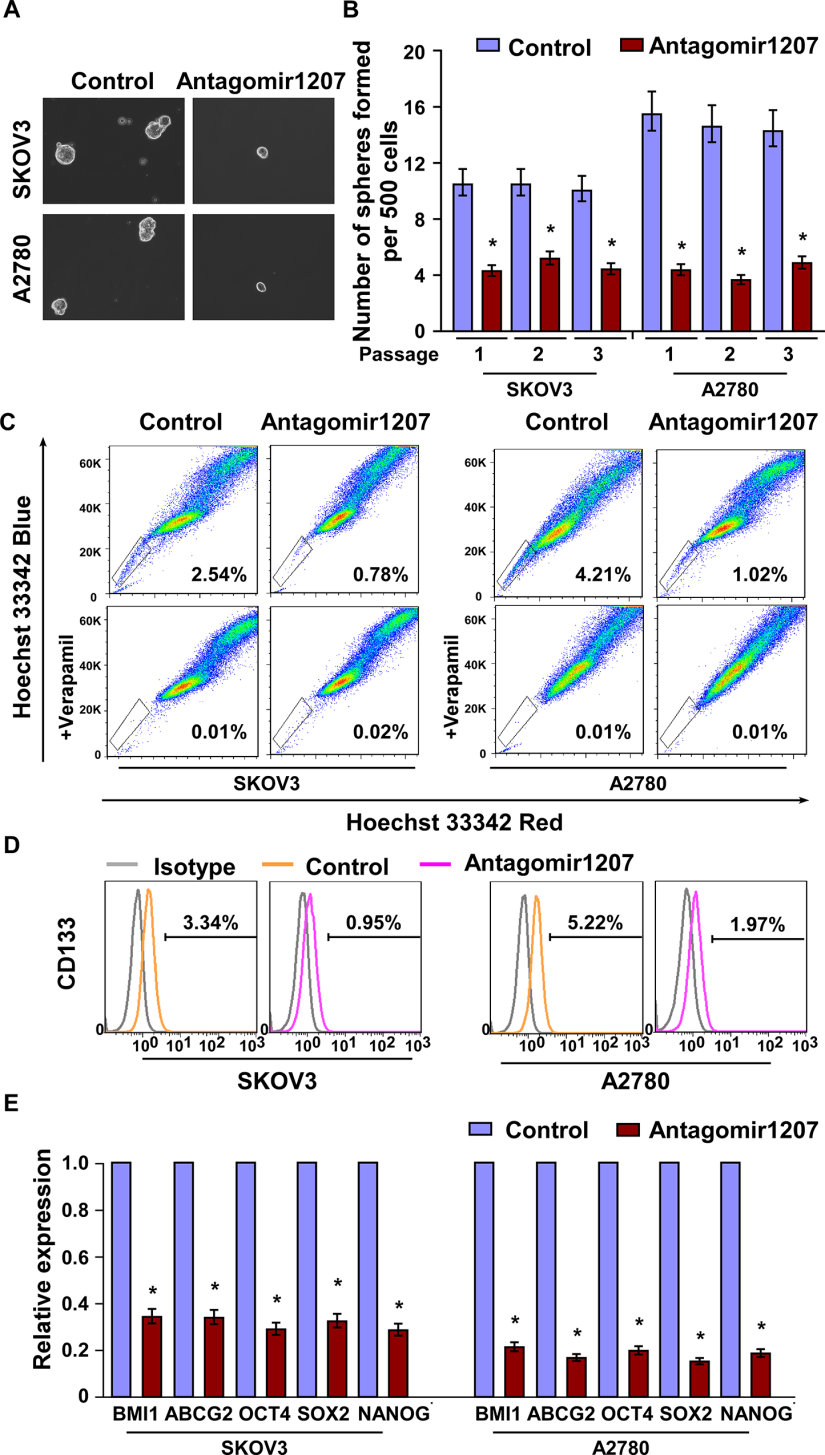
MiR-1207 inhibition reduces stem cell–like traits in ovarian cancer **A–B.** Representative micrographs (A) and quantification (B) of tumor sphere formation in antagomir-1207 or control cells. Scale bar, 100 μm. **C–D.** Effects of antagomir-1207 or control cells on the distribution of (C) SP^+^ and (D) CD133^+^ cells determined using flow cytometric analysis. **E.** Real-time PCR of the mRNA expression of pluripotency-associated markers in antagomir-1207 or control cells. Transcript levels were normalized to *GAPDH* expression. Error bars represent the mean ± SD of three independent experiments. *P < 0.05.

The effect of miR-1207 downregulation on the stem cell -like traits of ovarian cancer cells was further examined. AntagomiR-1207, an antisense-based specific inhibitor against miR-1207, was utilized to specifically silence endogenous miR-1207 (Supplementary Figure 1B). As shown in Figure 3A and 3B, the tumor sphere formation assay revealed that fewer and smaller spheres were formed by the miR-1207-silenced ovarian cancer cells. Consistently, the percentage of SP^+^ and CD133^+^ subpopulations was significantly decreased in antagomiR-1207 cells compared with control cells (Figure 3C–3D). Furthermore, miR-1207 inhibition significantly reduced the mRNA expression of BMI1, ABCG2, OCT4, SOX2 and NANOG (Figure 3E). Thus, our experiments indicate that silencing endogenous miR-1207 might suppress ovarian CSC-like traits.

### Upregulation of miR-1207 enhances tumourigenicity of ovarian cancer cells *in vivo*

**Figure 4:**
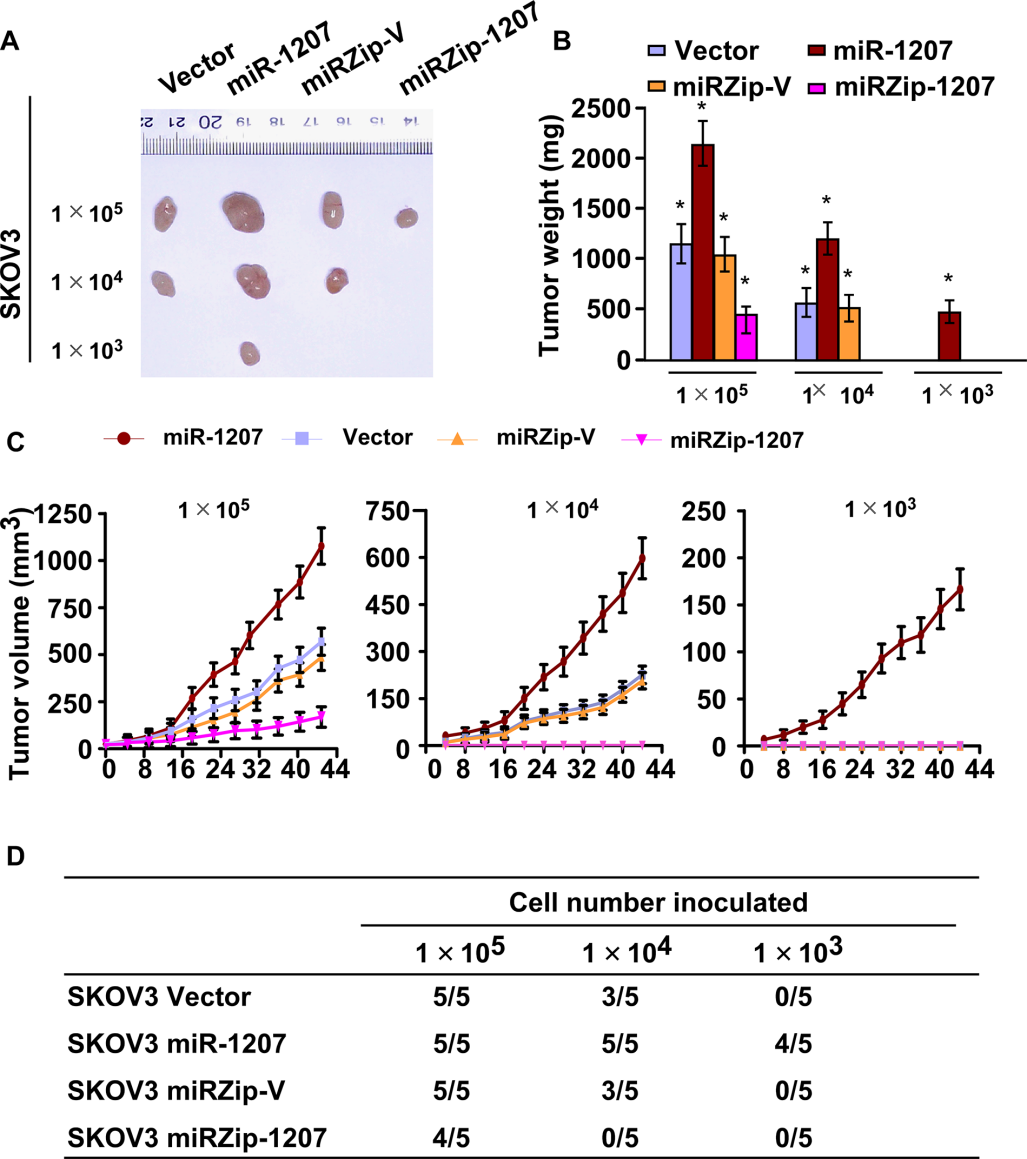
MiR-1207 enhances ovarian cancer cell tumorigenicity *in vivo* **A.** Xenograft model in nude mice. Tumors formed by miR-1207 overexpression SKOV3 cells were larger than the vector control tumors in the 1 × 10^5^ cells group. Conversely, tumors formed by antagomir-1207 cells were smaller than tumors formed by control cells. Only miR-1207 overexpression cells formed tumors following the implantation of 1 × 10^3^ cells. Representative images of the tumors are shown. **B.** Histograms showing the mean tumor weights of each group (*n* = 5 per group). *P < 0.05. **C.** Growth curves of tumor formation after implantation. Mean tumor volumes are plotted. **D.** Tumor formation initiated by SKOV3 cells in BALB/C nude mice.

In an effort to further confirm the effect of miR-1207 on ovarian cancer cell stemness, we subcutaneously inoculated different numbers of miR-1207-deregulated cells mixed with Matrigel into non-obese diabetic/severe combined immunodeficient (NOD/SCID) mice (Supplementary Figure S2A). As shown in Figure 4A–4C, the tumors formed by miR-1207-transduced ovarian cancer cells, at both amount of 1 × 10^5^, 1 × 10^4^ or 1 × 10^3^ cells, were dramatically larger than the vector control tumors. Conversely, miRZip-1207 cells formed much smaller tumors and presented lower rates of tumorigenesis (Figure 4A–4C). Importantly, only miR-1207-transduced cells could form tumors when 1 × 10^3^ cells were implanted (Figure 4D). These results indicate that miR-1207 strongly promotes ovarian cancer tumorigenicity *in vivo*.

### MiR-1207 directly targets multiple negative regulators of Wnt/β-catenin signaling

**Figure 5:**
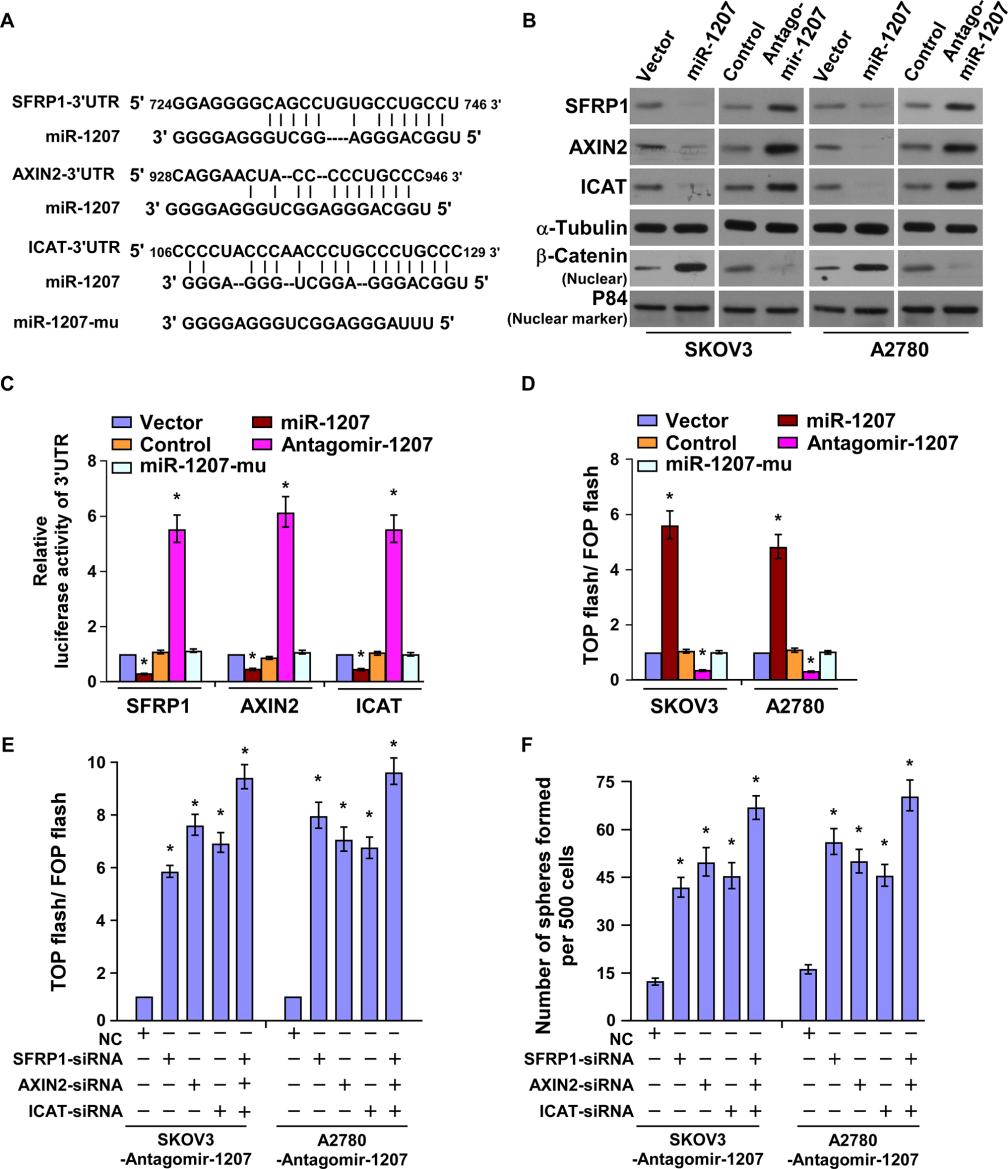
MiR-1207 directly targets SFRP1, Axin2, and ICAT **A.** Predicted miR-1207 target sequences in the 3′ UTRs of *SFRP1*, *AXIN2*, *ICAT*, and mutant containing three altered nucleotides in the miR-1207 seed sequence (miR-1207-mu). **B.** Western blot of SFRP1, Axin2, ICAT, and nuclear β-catenin in the cells. α-Tubulin served as the loading control. **C.** Luciferase activity of the 3′ UTRs of the targeted genes. Reporter activity detected was normalized to *Renilla* luciferase activity. **D.** Cells transfected with TOP-flash/FOP-flash and *Renilla* pRL-TK plasmids were subjected to dual luciferase assays 48 hours after transfection. **E.** Luciferase activity of TOP-flash/FOP-flash. **F.** Quantification of tumor sphere cells with specific small interfering RNA (siRNA) transfection. Bars represent the mean ± SD of three independent experiments. *P < 0.05.

By employing publicly available algorithms (TargetScan6.2 and miRanda), we found that three negative regulators of Wnt/β-catenin signaling, including SFRP1, AXIN2 and ICAT, might be miR-1207 targeting genes (Figure 5A). Western blotting analysis revealed that the expression levels of SFRP1, AXIN2 and ICAT were reduced in miR-1207-overexpressing cells, whereas miR-1207 inhibition increased the expression levels of these proteins (Figure 5B). Additionally, miR-1207 overexpression significantly repressed the luciferase activity of the 3′-untranslated region (3′-UTR) of SFRP1, AXIN2 and ICAT, whereas inhibition of endogenous miR-1207 increased the luciferase activity (Figure 5C). However, the miR-1207 mutant had no inhibitory effect on the luciferase activity of the three transcripts. Collectively, our results indicate that SFRP1, AXIN2 and ICAT are *bona fide* targets of miR-1207.

### MiR-1207 activates Wnt/β-catenin signaling

Since Wnt/β-catenin signaling pathway has been demonstrated to play important roles in the ovarian cancer progression and regulation of ovarian CSC-like traits [27], we therefore investigated whether miR-1207 regulates Wnt/β-catenin signaling in ovarian cancer cells. As shown in Figure 5A, overexpression of miR-1207 significantly increased, whereas inhibition of miR-1207 decreased, the luciferase activity of the TOP flash/ FOP flash reporter. However, the miR-1207 mutant did not show any effect on TOP flash/ FOP flash luciferase activity (Figure 5D). The downstream genes of Wnt/β-catenin signaling, such as C-myc, Cyclin D1 and LEF1, were also significantly upregulated in miR-1207-transduced cells but reduced in miR-1207-silenced cells (Supplementary Figure S3A). Furthermore, Western blotting proved that overexpressing miR-1207 induced, while inhibiting miR-1207 reduced, the nuclear expression of β-catenin (Figure 5B). These results indicate that miR-1207 activates Wnt/β-catenin signaling.

Silencing SFRP1, AXIN2, and ICAT potently antagonized the inhibitory effect of miR-1207-mediated Wnt/ β-catenin activity (Supplementary Figure S3B and Figure 5E), demonstrating that SFRP1, AXIN2, and ICAT are functionally relevant effectors of miR-1207 in activation of Wnt/β-catenin signaling. Furthermore, we found that the repression efficacy of silencing miR-1207 on tumor sphere formation was dramatically abolished by downregulation of SFRP1, AXIN2 and ICAT (Figure 5F). These results demonstrate that SFRP1, AXIN2, and ICAT are important for miR-1207–induced stem cell-like traits in ovarian cancer cell lines.

### MiR-1207 is clinically correlated with Wnt/β-catenin signaling in ovarian cancer

**Figure 6:**
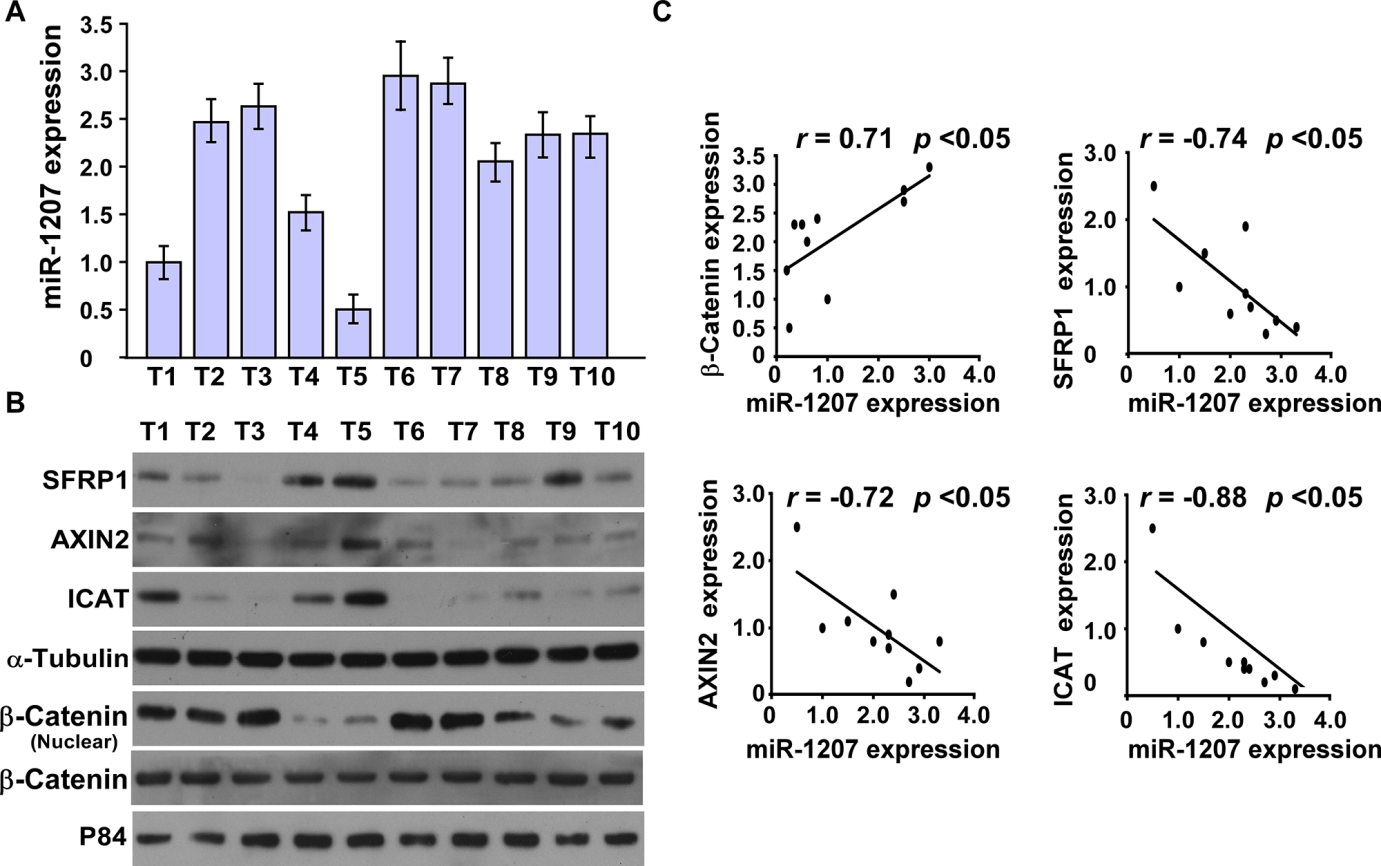
MiR-1207 levels were correlated with SFRP1, Axin2, ICAT, and nuclear β-catenin expression in ovarian cancer clinical tissues **A.** Real-time PCR of miR-1207 expression in 10 fresh ovarian cancer tissues (T) *U6* was used as the RNA loading control; miRNA levels were normalized to miR-1207 expression in Sample 1. Bars represent the mean ± SD of three independent experiments. **B.** Western blot of SFRP1, Axin2, ICAT, and nuclear β-catenin expression in the same 10 ovarian cancer tissues. α-Tubulin was used as the loading control. **C.** Correlation between miR-1207 levels and SFRP1, Axin2, ICAT, and nuclear β-catenin expression in ovarian cancer clinical tissues. The expression levels of SFRP1, AXIN2, ICAT and β-catenin, checked by western blotting analysis, were determined by densitometry. The ratio of first sample (SFRP1/α-tubulin, AXIN2/α-tubulin, ICAT/α-tubulin and β-catenin /α-tubulin) was considered as 1.0.

The clinical correlation between miR-1207 and Wnt/β-catenin signaling was further examined in 10 freshly collected clinical ovarian cancer samples. We found that miR-1207 expression was inversely correlated with the expression of SFRP1 (*r* = −0.74, *P* < 0.05), AXIN2 (*r* = −0.72, *P* < 0.05) and ICAT (*r* = −0.88, *P* < 0.05) but was strongly associated with nuclear β-catenin (*r* = 0.71, *P* < 0.05) (Figure 6A–6C). Collectively, these results further support the notion that miR-1207 upregulation promotes stem cell-like traits by activating Wnt/β-catenin signaling in ovarian cancer.

## DISCUSSION

In the present study, we found that miR-1207 was strongly overexpressed in ovarian cancer tissues, which was positively correlated with poor prognosis. Upregulation of miR-1207 induced, while downregulation of miR-1207 inhibited, ovarian cancer stem-like traits *in vitro*. By using *in vivo* model, we proved that the expression of miR-1207 was positively correlated with tumorigenicity. Furthermore, we demonstrated that miR-1207 enhanced ovarian cancer stem-like traits through directly targeting SFRP1, AXIN2 and ICAT, which are vital negative modulators of the Wnt/β-catenin pathway. Taken together, our results reveal a novel mechanism by which miR-1207 regulates ovarian CSC-like traits through activating the Wnt/β-catenin pathway, suggesting that miR-1207 might serve as a potential therapeutic target in ovarian cancer.

As a cohort of cells that possess the capacity to self-renew and to generate heterogeneous lineages of cancer cells, CSCs have been reported to play crucial roles in tumor initiation, metastasis, chemoresistance and recurrence [28, 29]. Furthermore, it has been reported that ovarian CSCs are associated with tumor recurrence, metastasis and invasion [30]. However, the underlying regulatory mechanism in ovarian CSCs remains largely unknown. In present study, we found that the overexpression of miR-1207 in ovarian cancer dramatically enhanced the ability of sphere formation and increased the population of SP^+^/CD133^+^ ovarian cancer cells *in vitro*. Moreover, overexpression of miR-1207 promoted, while suppression of miR-1207 inhibited ovarian cancer tumorigenicity *in vivo*. These results suggested that upregulation of miR-1207 might play an important role in promoting ovarian cancer stemness, which might provide a new insight into the CSCs modulation mechanisms.

The Wnt/β-catenin signaling pathway, a well-known pathway associated with progression and development of cancer, has been described as one of the most pivotal pathways involved in CSC maintenance [31]. In Cutaneous cancer, it has been reported that ablation of the β-catenin gene results in the loss of CSCs [32]. The tumor suppressive gene retinoic acid receptor responder 3(RARRES3) has been proved to inhibit cancer stem cell properties in breast cancer via targeting and modulating the acylation status of Wnt ligands and the co-receptor LRP6 [33]. Herein, we found that miR-1207 activated the Wnt/β-catenin pathway in different layers through direct downregulation of SFRP1, AXIN2, and ICAT, hence enhancing ovarian CSC-like traits, demonstrating a novel mechanism on how Wnt/β-catenin signaling modulates CSC properties and might be widespread application among other spectrum of cancers.

The dysregulation of SFRP1, AXIN2, and ICAT has been largely reported in numerous types of cancers [34–36]. For instance, downregulation of SFRP1 has been associated with tumor progression in breast cancer [37]. Reduced Axin protein expression is associated with poor prognosis in patients with esophagus squamous cell carcinoma [38]. Through competitive inhibition of the interaction between β-catenin and TCF, ICAT downregulation has been reported to increase cell motility and invasion in melanoma [39]. Our current study found that SFRP1, AXIN2, and ICAT were targeted and suppressed by miR-1207 in ovarian cancer, which promoted stem cell-like traits through highly activation of Wnt/β-catenin signaling. This study suggests a new regulatory mechanism of SFRP1, AXIN2, and ICAT, and also implies that the three genes play a significant role in mediating cancer stemness.

While little is known of miR-1207 in human cancers, a study by Chen L et al. suggested that miR-1207 suppresses gastric cancer growth and invasion by targeting telomerase reverse transcriptase. In our study, we found that miR-1207 was significantly overexpressed in ovarian cancer. Furthermore, ectopic expression of miR-1207 induced, whereas repression of endogenous miR-1207 reduced ovarian CSC-like trait via directly targeting SFRP1, AXIN2, and ICAT, three essential negative regulators of Wnt/β-catenin signaling pathway. More importantly, the *in vivo* model revealed that expression of miR-1207 was in positive relation with the rate of tumor formation and in inverse correlation with prognosis of ovarian cancer patients. These findings reveal that miR-1207 might act as a bio-marker for predicting the prognosis and progression of patients with ovarian cancer.

In summary, we found that elevated expression of miR-1207 promoted stem cell-like traits and tumorigenicity in ovarian cancer by simultaneously suppressing multiple inhibitors of Wnt/β-catenin signaling, namely SFRP1, AXIN2, and ICAT. We also found the inverse correlation between miR-1207 expression and prognosis of ovarian cancer patients. Therefore, these results uncover a novel molecular mechanism in which the Wnt/β-catenin pathway is constitutively activating in ovarian cancer and represent that miR-1207 may be a new prognostic biomarker and therapeutic target in ovarian cancer.

## MATERIALS AND METHODS

### Cell lines and cell culture

Human ovarian surface epithelial cells (HOSEpiC) was purchased from ScienCell Research Laboratories and cultured in ovarian epithelial cell medium. The ovarian cancer cell lines, including COV362, SKOV3, OV56, A2780, EFO-21, TOV-21G, TOV-112D and OV90 were grown in the DMEM medium (Invitrogen, Carlsbad, CA) supplemented with 10% fetal bovine serum (HyClone, Logan, UT).

### Patient information and tissue specimens

A total of 140 paraffin-embedded and archived ovarian cancer samples, which were histopathologically and clinically diagnosed at the Sun Yat-sen University Cancer Center from 2001 to 2006, were examined in this study. Prior patient consent and approval from the Institutional Research Ethics Committee were obtained for the use of these clinical materials for research purposes. Clinical information on the samples is summarized in Supplementary Table S1. All tumors were staged according to the International Federation of Gynaecology and Obstetrics standards (FIGO). Ten freshly collected ovarian cancer tissues and normal ovarian-adjacent tissues were frozen and stored in liquid nitrogen until further use, including FIGO stage I: 2, stage II: 4, stage III: 2 and stage IV: 2.

### Vectors, retroviral infection and transfection

The miR-1207 gene was PCR-amplified from genomic DNA and cloned into a pMSCV- puro retroviral vector. The miR-1207 anti-sense was cloned into miRZip plasmid purchased from System Biosciences and used according to previous report. [40] pMSCV-miR-1207 was cotransfected with the pIK packaging plasmid in HEK293T cells using the standard calcium phosphate transfection method. Thirty-six hours after the cotransfection, supernatants were collected and incubated with cells to be infected for 24 hours in the presence of polybrene (2.5 μg/ml). After infection, puromycin (0.5 μg/ml) was used to select stably transduced cells over a 10-day period [41]. The reporter plasmids containing wild-type (CCTTTGATC; TOP flash) or mutated (CCTTTGGCC; FOP flash) TCF/LEF DNA binding sites were purchased from Upstate Biotechnology (New York, USA). The 3′UTRs of SFRP1, AXIN2, and ICAT, respectively, were amplified and cloned downstream to the luciferase gene in a modified pGL3 control vector. Antagomir-1207 was purchased from RIBOBIO Company (Guangzhou, China).

### Primers and oligonucleotides

Cloning miR-1207: 5′- GCCAGATCTTGATTG ACTTACAG CCCAGTT-3′ and 5′-GCCGAA TTCCA CCTGTCTTTATTCCACCC-3′; Cloning SFRP1- 3′UTR-luci: 5′-GCCCCGCGG CTTGCCCTAACAACTCA-3′ and 5′-GCCCTGCAGTTT CCT TTGGGCGTTG-3′; Cloning AXIN2–3′UTR-luci: 5′-GCCCCGCGGATGTTTG CCGTGAGG A-3′ and 5′-GCCCTGCAGCCTGGGTCC ACATATTTAC-3′; Cloning ICAT-3′UTR-luci: 5′-G CCCC GCGGC GTTTATAGTTGAATTGG-3′ and 5′-GCCCTGC AGTTATTGGTACTAAAGCC T-3′. For depletion of SFRP1, AXIN2 and ICAT siRNA was synthesized and purified by RIBOBIO Company (Guangzhou, China).

### Luciferase reporter assay

Cells (3 × 10^4^) were seeded in triplicate in 24-well plates. 24-hours later, indicated luciferase reporter plasmids plus 3 ng pRL-TK Renilla plasmid were transfected into the cells using Lipofectamine 2000 Reagent (Life Technologies, USA). 48 hours after transfection, Dual Luciferase Reporter Assay (Promega, USA) was performed according to the manufacturer's instructions.

### Western blot analysis

Western blot analysis was performed using anti-SFRP1 (1:2000, Abcam, Cambridge, MA, USA); anti-AXIN2 (1:1000, Abcam, Cambridge, MA, USA), anti-ICAT (1:1000, Abcam, Cambridge, MA, USA); anti-β-catenin (1:1000, Abcam, Cambridge, MA, USA). To control sample loading, the blotting membranes were stripped and re-probed with an anti–α-tubulin or anti-p84 antibody (1:1000, Sigma, Saint Louis, MO, USA). Nuclear extracts were prepared using the Nuclear Extraction Kit (Active Motif), according to the manufacturer's instructions. Briefly, cells were collected in the PBS/phosphatase inhibitors solution and lysed in the Lysis Buffer, and then centrifuged for 5 minutes. Suspending nuclear pellet in 50 ul Complete Lysis Buffer, then incubating for 30 minutes on ice. Vortex the mixture for 30 seconds followed by centrifuge for 10 minutes at 14,000 g at 4°C, the collected nuclear extraction were store at −80°C for further examination. The expression levels of SFRP1, AXIN2, ICAT and β-catenin were determined by densitometry. The ratio of first sample (SFRP1/α-tubulin, AXIN2/α-tubulin, ICAT/α-tubulin and β-catenin /α-tubulin) was considered as 1.0.

### Animal studies

All experimental procedures were approved by the Institutional Animal Care and Use Committee (IACUC) of Sun Yat-sen University. The 6-week-old BALB/c-nu mice were randomly divided into three groups (*n* = 5 per group). Cells (1 × 10^5^, 1 × 10^4^, 1 × 10^3^) were inoculated subcutaneously together with Matrigel (final concentration of 25%) into the inguinal folds of the nude mice. Tumor volume was determined using an external caliper and calculated using the equation (L × W^2^)/2. The mice were sacrificed 35 days after inoculation and the tumors were excised and subjected to pathologic examination.

### Tumor sphere formation assays

Five hundred cells were seeded in 6-well ultra-low cluster plates and 10 or 20 cells were seeded in 24-well ultra-low cluster plates for 10 days. Spheres were cultured in DMEM/F12 serum-free medium (Invitrogen, USA) supplemented with 2% B27 (Invitrogen, USA), 20 ng/ml EGF, 20 ng/ml bFGF, and 5 μg/ml insulin (PeproTech, USA).

### Flow cytometric analysis

Cells were dissociated with trypsin and resuspended at 1 × 10^6^ cells/mL in DMEM containing 2% FBS, and then incubated at 37°C for 30 minutes with or without 100 μM verapamil (Sigma-Aldrich) to inhibit ATP-binding cassette (ABC) transporters. The cells were subsequently incubated for 90 minutes at 37°C with 5 μg/mL Hoechst 33342 (Sigma-Aldrich). Lastly, cells were incubated on ice for 10 minutes and washed with ice-cold phosphate-buffered saline (PBS) prior to flow cytometry analysis. Flow cytometric analysis or flow cytometric cell sorting was conducted using fluorescein isothiocyanate (FITC)-conjugated monocloncal mouse anti-human CD133 (Miltenyi Biotec GmbH, Germany). Samples were analyzed and sorted on BD FACSCanto II and FACSAria I, respectively (BD Biosciences, USA) with data analyzed using FlowJo software (Tree Star Inc, USA).

### Bioinformatics analysis

The following on-line software programs were used for bioinformatics analysis: TargetScan 6.2 (http://www.targetscan.org/); and miRanda (http://www.microrna.org/microrna/getGeneForm.do).

### Statistical analysis

All statistical analyses were carried out using SPSS 18.0 statistical software. The Kaplan-Meier method was used to establish survival curves, and the survival differences were compared using the log-rank test. Continuous data were compared using Student's 2-tailed *t*-test. In all cases, *P* < 0.05 was considered statistically significant.

## SUPPLEMENTARY MATERIAL FIGURES AND TABLES


