# Supplementary material for: MiR-1207 overexpression promotes cancer stem cell–like traits in ovarian cancer by activating the Wnt/β-catenin signaling pathway
Source: Oncotarget. 2015 Aug 17;6(30):28882–94. doi: 10.18632/oncotarget.4921 (PMC4745698; doi:10.18632/oncotarget.4921)
Supplement: Supplementary file 1 [file oncotarget-06-28882-s001.pdf]

SUPPLEMENTARY FIGURES AND TABLES

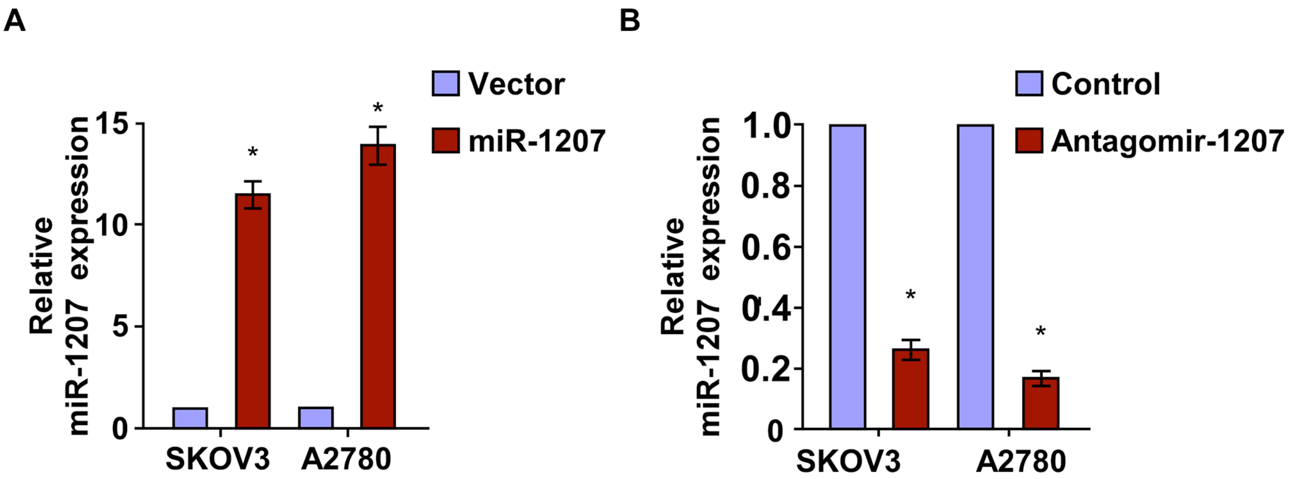

Supplementary Figure S1: Relative miR-1207 expression in A. miR-1207–transduced or B. miR-1207–silenced cells. Bars represent the mean ± SD of three independent experiments. *$P < 0.05$.

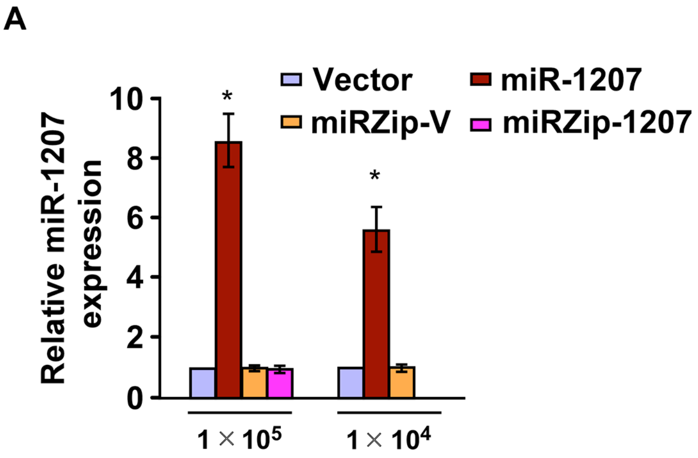

Supplementary Figure S2: Relative miR-1207 expression in the indicated xenograft tumors.

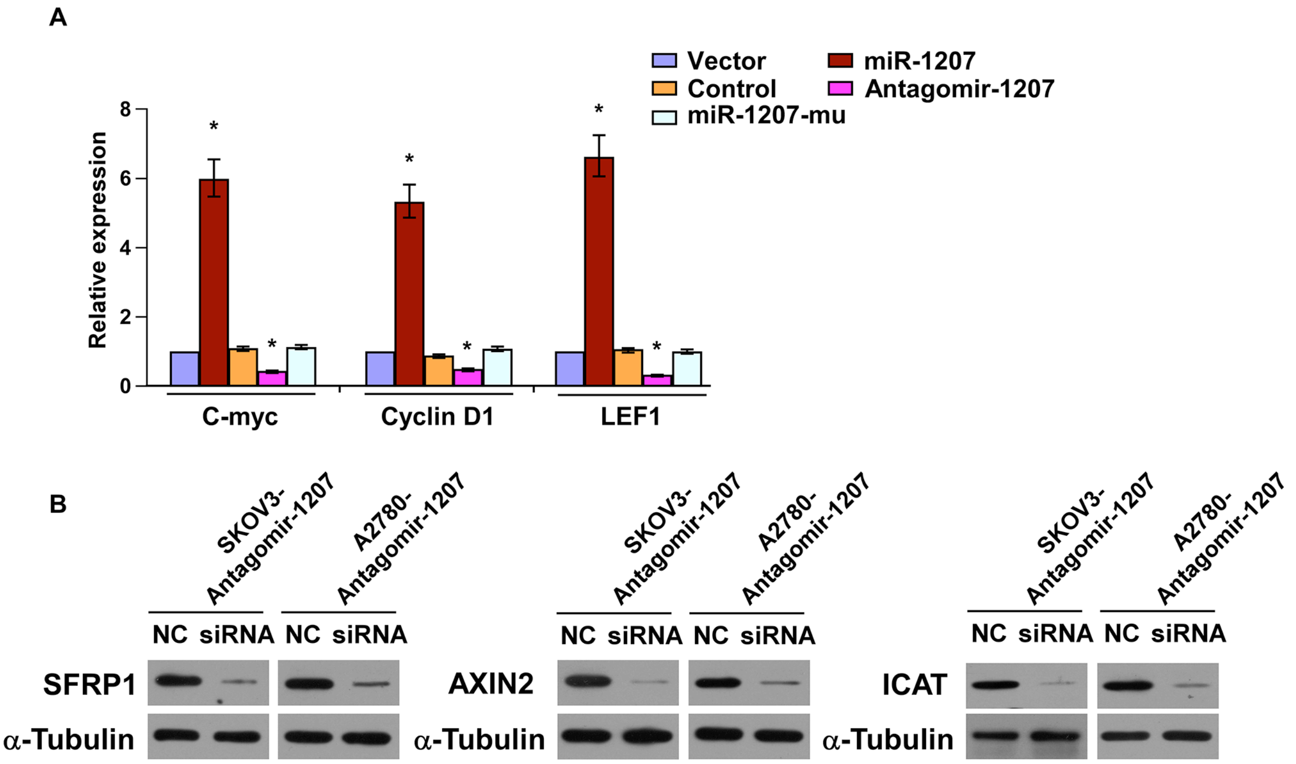

Supplementary Figure S3: A. Relative expression of c-myc, cyclin D1 and LEF1 in ovarian cancer cells with different expression level of miR-1207. **B.**Western blot of SFRP1, Axin2, and ICAT expression levels in antagomir-1207 cells transfected with SFRP1 siRNA (left), AXIN2 siRNA (middle), or ICAT siRNA (right). α-Tubulin served as the loading control. NC, negative control.

Supplementary Table S1: Clinicopathological characteristics and expression of miR-1207 in studied ovarian cancer patients

Factor	No.	(%)
**Age (years)**		
≤50	73	52.1
>50	67	47.9
**FIGO stage**		
I, II	48	34.3
III, IV	92	65.7
**Lymph node metastasis**		
None	88	62.9
Yes	52	37.1
**Vital status**		
Alive	62	44.3
Dead	78	55.7
**Expression of miR-1207**		
Low expression	70	50.0
High expression	70	50.0

Supplementary Table S2: Correlation between the clinicopathological features and expression of miR-1207

Patient characteristics		miR-1207 expression		*P*-value
		Low	High	
**Age (years)**	≤50	32	41	0.12
	>50	38	29	
**FIGO stage**	I, II	34	14	<0.001
	III, IV	36	56	
**Lymph node metastases**	None	52	36	<0.001
	Yes	18	34	
**Vital status**	Alive	41	21	<0.001
	Dead	29	49	
